# Evaluation of Resistance to Fescue Toxicosis in Purebred Angus Cattle Utilizing Animal Performance and Cytokine Response

**DOI:** 10.3390/toxins12120796

**Published:** 2020-12-14

**Authors:** Daniel H. Poole, Kyle J. Mayberry, McKayla Newsome, Rebecca K. Poole, Justine M Galliou, Piush Khanal, Matthew H. Poore, Nick V. L. Serão

**Affiliations:** 1 Department of Animal Science, North Carolina State University, Raleigh, NC 27695, USA; kmayberry@biltmore.com (K.J.M.); manewsom@ncsu.edu (M.N.); rkpoole@exchange.tamu.edu (R.K.P.); justine.galliou@wsu.edu (J.M.G.); pkhanal2@ncsu.edu (P.K.); mhpoore@ncsu.edu (M.H.P.); serao@iastate.edu (N.V.L.S.); 2 Director of Agriculture, The Biltmore Company, Asheville, NC 28803, USA; 3 Department of Animal Science, Texas A&M University, College Station, TX 77843, USA; 4 Department of Animal Science, Iowa State University, Ames, IA 50011, USA

**Keywords:** fescue toxicosis, genetic tolerance, cytokines, cow productivity

## Abstract

Fescue toxicosis is a multifaceted syndrome common in cattle grazing endophyte-infected tall fescue; however, varying symptomatic responses potentially imply genetic tolerance to the syndrome. It was hypothesized that a subpopulation of animals within a herd would develop tolerance to ergot alkaloid toxicity. Therefore, the goals of this study were to develop selection criteria to identify tolerant and susceptible animals within a herd based on animal performance, and then examine responsive phenotypic and cytokine profiles to fescue toxicosis. Angus cows grazed endophyte-infected tall fescue at two locations for 13 weeks starting in mid-April 2016. Forage measurements were collected to evaluate ergot alkaloid exposure during the study. A post hoc analysis of animal performance was utilized to designate cattle into either tolerant or susceptible groups, and weekly physiological measurements and blood samples were collected to evaluate responses to chronic exposure to endophyte-infected tall fescue. Findings from this study support the proposed fescue toxicosis selection method formulated herein, could accurately distinguish between tolerant and susceptible animals based on the performance parameters in cattle chronically exposed to ergot alkaloids, and provides evidence to warrant additional analysis to examine the impact of ergot alkaloids on immune responsiveness in cattle experiencing fescue toxicosis.

## 1. Introduction

Fescue toxicosis, resulting from consumption of ergot alkaloids commonly found in endophyte (*Epichloë coenophiala*)-infected tall fescue (*Lolium arundinaceum* Schreb. Darbysh), significantly impacts livestock health and production globally. Direct exposure to ergot alkaloids from tall fescue and other grass species occurs in production systems that heavily rely on grazing, such as in cow calf and stocker programs in the United States, New Zealand, and Australia; whereas importation of feedstuff has led to ergot alkaloid-induced effects in several Asian countries, including Japan and Korea [1,2,3]. Due to the agronomic benefits resulting from symbiosis with the endophyte, including stand persistence and drought tolerance, it is expected that endophyte-infected tall fescue will become the primary forage across more growing zones as the global climate warms. McCulley et al. [4] recently reported that warming air temperatures (+3 °C, day and night, year-round) significantly increased concentrations of ergovaline and total ergot alkaloids found in endophyte-infected tall fescue, which may exacerbate fescue toxicosis in future years. Complex plant–fungus–environment interactions exist due to variation in endophyte infection rate, alkaloid production (125 to 5000 µg/Kg), and the animal’s voluntary intake [4], all of which can lead to high variation in individual animal responses. Individual variation in response to fescue toxicosis might be, in part, due to host genetics. Galliou et al. [5] tested the association between genotypes from a commercial genetic test for fescue toxicosis and performance in pregnant Angus cows. These authors found significant associations with growth, hair shedding, and calf weaning weight. Altogether, these pose a major challenge to understanding the underlying mechanisms of action responsible for the decreases in livestock productivity. While advancements have been made to better understand the mechanisms associated with fescue toxicosis symptoms, such as focusing on improving growth and reproductive performance of beef cattle grazing endophyte-infected tall fescue, a greater understanding is needed to identify markers for tolerance to fescue toxicosis, which is critical for the sustainability of the global beef industry. 

Tall fescue, and more specifically the cultivar Kentucky 31, is the most abundant and economically important cool-season perennial grass in U.S. agriculture. Approximately 8.5 million head of beef cattle reside in southeast or central U.S., where fescue is the predominant forage available, most of which contains the endophyte that causes toxicosis in grazing livestock. While precise data on the rate and composition of ergot alkaloid intake in grazing cattle is limited, the symptoms of this multifaceted disease range from acute outbreaks of gangrenous ergotism to more subtle and chronic decreases in livestock productivity characterized by decreased feed intake and growth performance, compromised reproduction (pregnancy and calving rates), elevated body temperature, and reduced blood flow to the extremities, as reviewed by Strickland et al. [1]. While the gangrenous ergotism is rare and most likely due to acute exposure to very high concentrations of ergot alkaloids, combined with cold temperatures, the decreased livestock health, welfare, and productivity is expected as a result of chronic exposure to ergot alkaloids. 

While there have been extensive efforts to develop an innovative and strategic pasture and grazing management system to convert toxic tall fescue to the non-toxic “Novel Endophyte Tall Fescue”, many farmers have exercised some caution and hesitation to adopt the technology. This appears to be due to lack of understanding of how to renovate, expense, time, challenging terrain, and lack of feed resources to utilize while the new forage is being established. Essentially, the goal of converting all of the toxic fescue to novel endophyte tall fescue is likely impractical and highly unlikely. Therefore, there has been concurrent interest in identifying ways that producers can select animals based on their tolerance to the adverse effects of fescue toxicosis. As previously stated, animals suffering from fescue toxicosis exhibit decreased growth capability, poor reproductive performance, and have a lack of adequate heat tolerance at least in part due to their retained winter hair coat. Focusing on growth performance selection parameters and the animal’s cytokine response to the toxin could be utilized to identify animals that are tolerant to the effects of fescue toxicosis. Therefore, the goals of this study were to develop selection criteria to identify tolerant and susceptible animals based on phenotypic animal performance traits, to evaluate how our criteria matches with the information from a commercial test for fescue toxicosis, and then examine responsive cytokine profiles to identify beef cattle displaying tolerance to fescue toxicosis. 

## 2. Results

### 2.1. Changes in Forage Characteristics and Ergot Alkaloid Concentration 

Forage fractions during period 1 (P-1) of the experiment were comprised of 72.5 and 72.9 percent fescue at the Butner Beef Cattle Field Lab (BBCFL) and Upper Piedmont Research Station (UPRS) locations, respectively. The percentage of fescue available to these animals during period 2 (P-2) decreased to 56.0 and 63.3 percent at BBCFL and UPRS, respectively (Table 1). Overall, animals that grazed pastures located at BBCFL were exposed to a lower percentage of fescue compared to UPRS. Additionally, tiller samples to determine the endophyte infection rate identified that the BBCFL pastures had a lower infection rate compared to the UPRS pastures throughout the course of the trial, 80.8 compared to 86.3 percent, respectively (Table 1). As a result of the differences in infection rate and the percent of tall fescue in the sward, ergovaline concentrations differed between these locations (Table 1). The highest concentrations of ergovaline were observed in P-1 for both locations (263.3 μg/kg for BBCFL and 436.7 μg/kg for UPRS) and decreased during P-2 (106.7 μg/kg for BBCFL and 196.7 μg/kg for UPRS) by the end of the trial (Table 1). During P-2 at UPRS, ergosine (333 μg/Kg), ergotamine (175 μg/Kg), ergocornine (53 μg/Kg), ergocriptine (150 μg/Kg), and ergocristine (48 μg/Kg) contributed to total ergot alkaloids. These alkaloids are characteristic of Claviceps purpurea [6], which can commonly infect tall fescue and other grasses. While the concentration of ergovaline decreased during P-2, these concentrations (along with the *C. purpurea* alkaloids at UPRS) were still high enough to elicit many of the negative symptoms associated with fescue toxicosis [7,8]. Lastly, the nutritive value and mineral composition of the tall fescue pastures utilized for this study are presented in Supplementary Table S1. Total digestible nutrients, crude protein (CP), neutral detergent fiber (NDF), and acid detergent fiber (ADF) were not different between the two locations and did not change over the course of the trial.

### 2.2. Distribution of T-Snip Genotypes Across FTSM Selected Animals

The expected proportion of T-Snip genotypes between the two FTSM groups can be see in Table 2. There is a tendency for a different distribution of T-Snip genotypes between EI-TOL and EI-SUS animals (*p* = 0.089). There was a statistical difference (*p* < 0.05) for the proportions of T-Snip genotypes 2 and 3 between EI-TOL and EI-SUS animals. EI-TOL animals had a lower proportion of genotype 2 than EI-SUS animals (0.1 ± 0.07 versus 0.368 ± 0.11), whereas the opposite was found for T-Snip genotype 3 (0.6 ± 0.11 versus 0.316 ± 0.11). There were no differences in the proportion of T-Snip genotypes 1 and 4 between these animals (*p* > 0.05). These results indicated that EI-TOL animals had overall greater frequency of greater T-Snip genotypes than EI-SUS animals. 

### 2.3. Phenotypic Variables of Animal Performance 

**Figure 1:**
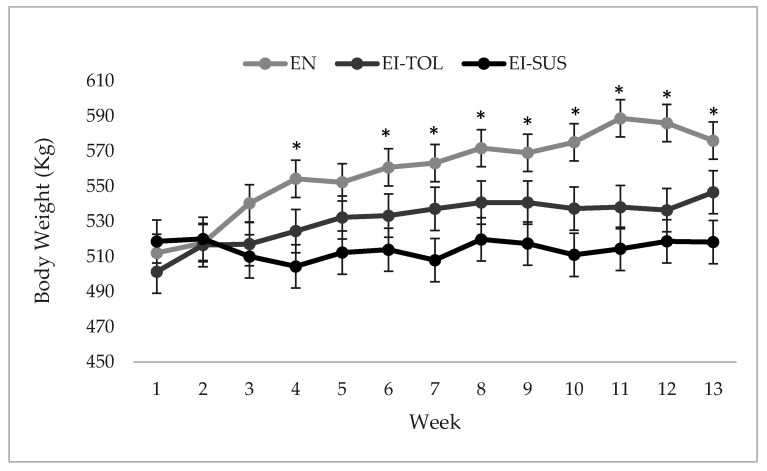
Body weight by treatment over time (weeks): Cows (*n* = 27) grazing on novel endophyte fescue pastures (EN, non-toxic) had greater body weights compared to grazing endophyte infected (EI, toxic) fescue pastures. Tolerant animals (EI-TOL) had greater average body weight compared to susceptible (EI-SUS) animals, determined by FTSM; (*n* = 40). The model was significant by location and treatment X location (trt, loc, trt X loc *p*-values: *p* < 0.0001).

Animal performance is negatively impacted during ergot alkaloid exposure, thus, the focus of this study was to identify tolerant and susceptible animals within an exposed population; however, direct comparisons of performance parameters to animals not exposed to ergot alkaloids demonstrated the severity of exposure throughout the trial. Cattle grazing novel endophyte fescue pastures (EN, non-toxic) had greater body weights compared to animals grazing endophyte-infected (EI, toxic) fescue pastures (*p* < 0.0001; Figure 1). By comparison, animals deemed tolerant (EI-TOL) to ergot alkaloid exposure displayed a positive weight gain compared to animals deemed susceptible (EI-SUS) to ergot alkaloid exposure (Figure 1; Table 3; *p* < 0.05). Additionally, cattle grazing novel endophyte fescue pastures had a greater average daily gain (ADG; 0.703 kg/d) and body condition score (BCS; 6.1) compared to EI-TOL and EI-SUS animals (0.497 and −0.003 kg/d, respectively, for ADG and 5.5 and 5.6, respectively, for BCS; Table 3; *p* < 0.0001). Throughout the course of the study, EN and EI-TOL animals displayed similar hair coat scores (HCS) and were significantly less dense compared to EI-SUS animals (Table 3; *p* < 0.0001). Furthermore, EN animals were better able to shed their hair throughout the trial period compared to EI-TOL and EI-SUS animals (1.99 versus 2.36 and 2.64, respectively; Table 3; *p* < 0.0001). Although within the normal body temperature range for cattle, EN animals were cooler compared to EI-TOL and EI-SUS animals as indicated by rectal temperatures (Table 3; *p* < 0.0001). 

The average daily gain was significantly greater in EI-TOL animals during P-1 and over the entire study (ES) compared to EI-SUS animals (Table 4); however, no difference in ADG was observed during P-2 (Table 4), which could be reflective of the change in ergovaline concentrations observed in the fescue pastures between P-1 and P-2 of the trial. In addition to ADG, EI-TOL animals displayed a greater positive change in BCS during P-1, P-2, and throughout the entire study compared to the EI-SUS animals (Table 4; *p* < 0.05). Throughout the course of the study, EI-TOL animals displayed greater change in HCS and HSS in P-1 and P-2 compared to EI-SUS animals (Table 4; *p* < 0.05), with the greatest change occurring in P-1 as the temperature and humidity increased. Thus, EI-TOL animals were able to alter their hair coat density and length earlier in the year when ergot alkaloid concentrations were elevated (P-1), reducing the negative impact of fescue toxicosis on animal performance. Interestingly, no differences were observed when examining the change in HCS or HHS between EI-TOL and EI-SUS animals over the entire study (Table 4; *p* > 0.05). When examining differences in HCS and HSS between P-1 and P-2, there was an increase in hair shedding in the EI-SUS animals in P-2, which could be attributed to the decrease in ergot alkaloid concentration in the fescue pastures, in combination with the fact that elevated temperatures and humidity permitted these animals to display similar hair characteristics to that of the EI-TOL animals. However, no differences were observed in the change in body temperatures during P-1, P-2, or over the entire study (Table 4; *p* > 0.05). 

### 2.4. Cytokine Response and Hormone Profiles in FTSM Selected Animals

While performance parameters can be directly selected, changes within the peripheral concentrations of growth factors (FGF1, FGF2, IGF1, and VEGFA); chemokines (CCL2, CCL4, CXCL9, CXCL10); cytokine receptor antagonists (IL36RN, GPRASP-1, NCAM1); anti-inflammatory (IL4 and IL13); and pro-inflammatory (IFNAR1, IFNG, IL1A, IL15, IL2, IL21 and TNF) cytokines, as well as reproductive hormones (prolactin and progesterone), in response to ergot alkaloid exposure would potentially lead to a novel marker for identifying animals resistant to fescue toxicosis.

**Figure 2:**
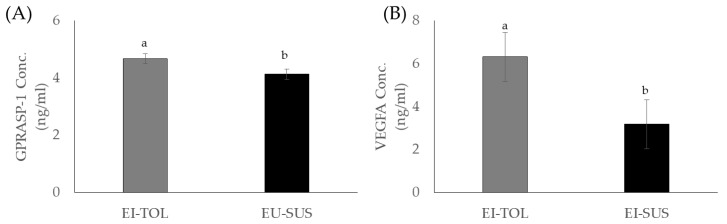
EI-TOL animals (*n* = 20) had greater concentrations of GPRASP-1 (**A**) and VEGFA (**B**) in peripheral blood compared to EI-SUS animals (*n* = 20) when chronically exposed to endophyte-infected tall fescue (*p* < 0.05).

Of the growth factors, vascular endothelial growth factor A (VEGFA) concentrations were greater in EI-TOL animals compared to EI-SUS animals (6.3 ± 1.1 versus 3.2 ± 1.1 ng/mL, respectively; Figure 2B; *p* = 0.0444). No differences were observed in VEGFA concentrations between locations or over the course of the trial (Table 5; *p* > 0.05). No differences were observed in FGF1, FGF2, or IGF1 concentrations between EI-TOL and EI-SUS animals over the course of the trial (Table 5; *p* > 0.05). Fibroblast growth factor 1, but not FGF2 or IGF1, concentrations tended to be greater in animals at UPRS compared animals at BBCFL (328.6 ± 81.6 versus 62.4 ± 81.6 ng/mL, respectively; Table 5; *p* = 0.0793). 

No differences were observed in chemokines, CCL2, CXCL9, or CXCL10 concentrations between EI-TOL and EI-SUS animals over the course of the trial (Table 5; *p* > 0.05). Macrophage inflammatory protein-1β (CCL4) concentrations were not difference between EI-TOL and EI-SUS animals (*p* > 0.05), however, CCL4 concentrations were greater in animals at UPRS compared to animals at BBCFL (161.0 ± 24.0 versus 100.7 ± 24.0 ng/mL, respectively; Table 5; *p* = 0.0793) and increased over the course of the trial (10.7 ± 98.9, 202.6 ± 98.9, and 373.1 ± 98.9, for weeks 1, 7, and 13, respectively; Table 5; *p*= 0.0428). Furthermore, CXCL9 and CXCL10 concentrations tended to be greater in animals at UPRS compared animals at BBCFL (132.5 ± 28.5 versus 58.9 ± 28.5 ng/mL, respectively, for CXCL9 and 73.8 ± 11.0 versus 46.9 ± 11.0 ng/mL, respectively, for CXCL10; Table 5).

G Protein-Coupled Receptor Associated Sorting Protein 1 (GPRASP-1) concentrations were greater in EI-TOL animals compared to EI-SUS animals (4.7 ± 0.2 versus 4.1 ± 0.2 ng/mL, respectively; Figure 2A; *p* = 0.0343). No differences were observed in GPRASP-1 concentrations between locations (Table 5; *p* > 0.05), however, GPRASP-1 concentrations were greatest at the end of P-1, compared to the start and end of the trial (5.0 ± 0.2 versus 4.1 ± 0.2 for weeks 1 and 13, Table 5; *p* = 0.0033). No differences were observed in IL36RN or NCAM1 concentrations between EI-TOL and EI-SUS animals over the course of the trial (Table 5; *p* > 0.05). However, IL36RN and NCAM1 concentrations were greater in animals at UPRS compared animals at BBCFL (333.4 ± 57.4 versus 97.1 ± 57.4 ng/mL, respectively, for IL36RN and 35.5 ± 1.5 versus 31.2 ± 1.5 ng/mL, respectively, for NCAM1; Table 5). 

No differences were observed in anti-inflammatory cytokine, IL4, or IL13 concentrations between EI-TOL and EI-SUS animals over the course of the trial (Table 6; *p* > 0.05). However, IL4 and IL13 concentrations tended to be greater in animals at UPRS compared animals at BBCFL (218.8 ± 56.2 versus 83.3 ± 56.2 ng/mL, respectively, for IL4 and 12.5 ± 2.2 versus 6.9 ± 2.2 ng/mL, respectively, for IL13; Table 6). No differences were observed in pro-inflammatory cytokine, IFNG, IL1A, IL15, and IL2 concentrations between EI-TOL and EI-SUS animals between locations or over the course of the trial (Table 6; *p* > 0.05). While no differences were observed in IFNAR1 concentrations between EI-TOL and EI-SUS animals or between locations, IFNAR1 concentrations increased over the course of the trial (41.2 ± 62.4, 165.4 ± 62.4, and 411.9 ± 62.4, for weeks 1, 7, and 13, respectively; Table 6; *p* = 0.0001). Additionally, no differences were observed in IL21 or TNF concentrations between EI-TOL and EI-SUS animals over the course of the trial (Table 6; *p* > 0.05), but concentrations of these cytokines tended to be greater in animals at UPRS compared animals at BBCFL (135.3 ± 24.6 versus 73.7 ± 24.6 ng/mL, respectively, for IL21and 149.4 ± 29.6 versus 67.2 ± 29.6 ng/mL, respectively, for TNF; Table 6).

Of the reproductive hormones examined, prolactin (PRL) concentrations tended to be greater in EI-SUS animals compared to EI-TOL animals (138.4 ± 16.0 versus 98.3 ± 16.0 ng/mL, respectively; Table 6; *p* = 0.0787). Additionally, PRL concentrations tended to be greater in animals at UPRS compared animals at BBCFL (137.6 ± 15.8 versus 99.1 ± 15.8 ng/mL, respectively; Table 6), and was significantly less at the start of the trial compared to weeks 7 and 13 of the trial (64.0 ± 19.4 versus 163.4 ± 19.4 and 127.6 ± 19.4 for weeks 7 and 13, respectively; Table 6; *p* = 0.0019); progesterone (P4) concentrations tended to be greater in EI-TOL animals compared to EI-SUS animals (3.3 ± 0.14 versus 3.0 ± 0.14 ng/mL, respectively; Table 6; *p* = 0.0895). Additionally, P4 concentrations were greater in animals at BBCFL compared animals at UPRS (3.4 ± 1.4 versus 2.8 ± 1.4 ng/mL, respectively; Table 6) and was significantly less at week seven of the trial compared to weeks 1 and 13 of the trial (2.2 ± 0.3 versus 3.5 ± 0.3 and 2.8 ± 0.2 for weeks 1 and 13, respectively; Table 6; *p* = 0.0003).

## 3. Discussion

Due to the extensive use of endophyte-infected tall fescue across the globe, the goals of this study were to develop selection criteria to identify tolerant and susceptible animals based on phenotypic animal performance traits, and then examine responsive cytokine profiles to identify beef cattle displaying tolerance to fescue toxicosis. Several studies described breed differences in response to ergot alkaloids, in which breeds such as Senepol and Brahman, which are better able to handle the heat and humidity, outperformed British breeds such as Angus and Hereford [8,10,11,12] when exposed to toxic tall fescue. These breeds have been used in crossbreeding programs to address fescue toxicosis, but it is unclear if their apparent tolerance is to fescue or adaptation to elevated heat and humidity. However, genetic progress is obtained within the breed, and the identification of genetic variation in animals from the same breed should be exploited to allow for selection for improved response (e.g., tolerance) to fescue toxicosis. Gray et al. [13] examined Angus cattle performance in North Carolina and Mississippi on endophyte-infected fescue pasture. These researchers observed genetic variation for hair coat shedding, indicating that Angus cattle that shed their winter hair coat earlier in the year may be more heat tolerant.

At the genomic level, genes regulating prolactin production have been targeted to identify genetic markers for tolerance to fescue toxicosis. Campbell et al. [14] recently identified a single nucleotide polymorphism (SNP) within the dopamine receptor D2 gene that was associated with variation in calving rates when grazing endophyte-infected tall fescue. In addition, using part of the same data in this study, Galliou et al. [5] showed that a commercial genetic test for fescue toxicosis, T-Snip (AgBotanica, LCC, Columbia, MO, USA), is associated with growth, hair shedding, and calf weaning weight in pregnant Angus cows. With the available T-Snip data, we tested whether the distribution of T-Snip genotypes changed between EI-TOL and EI-SUS animals in our study. According to the company’s instructions, T-Snip genotypes range from 1 to 5, with levels of tolerance to fescue toxicosis increasing with the value of the genotype. Although both groups of animals showed T-Snip genotypes 1 to 4, there was a greater proportion of genotype 3 in EI-TOL than in EI-SUS. Similarly, there was a greater proportion of genotype 2 in EI-SUS than in EI-TOL. Therefore, the expected genetic values based on this commercial genetic test was greater in EI-TOL than in EI-SUS. Hence, although the selection of animals in this study was fully based on their phenotypic performance, results from T-Snip genotypes further support that this selection method was able to identify animals with contrasting tolerance to fescue toxicosis. It is important to note that this commercial genetic test does not have a perfect accuracy to predict with phenotypic performance in animals during fescue toxicosis [5]. Therefore, the presence of animals with lower and greater genotype values in EI-TOL and EI-SUS groups, respectively, was expected. Considering these factors, developing a selection method based on the phenotypic performance parameter in cattle from a single breed source in controlled experiments would aid in identifying animals that are potentially tolerant or susceptible to ergot alkaloids. 

A major challenge with studying this multifaceted syndrome is that endophyte production of ergot alkaloids varies with season and plant maturity, and contributes to variation in ergot alkaloid intake by grazing cattle. Total ergot alkaloids or ergovaline, the most abundant alkaloid, has been measured in fescue to describe its potential toxicity. Concentrations of ergovaline increase from 250 to 500 µg/Kg in leaf blades and from 500 to 1300 µg/Kg in leaf sheaths from April to May. Seed heads contain the greatest concentration of toxins, and reach concentrations as high as 5000 µg/Kg in June. Ergovaline concentrations decline in August and increase again during fall regrowth [9]. Total ergot alkaloid concentration displays the same seasonal changes as ergovaline [15]. The concentration of total ergot alkaloids and ergovaline declines by 81% to 85%, respectively, from December to March in stockpiled fescue (accumulated during the growing season for grazing during dormancy; [16,17]). There were differences in the percentage of fescue and endophyte infection rate between locations used in this study, which then led to differences in ergovaline concentrations between locations, as well as variation in ergovaline concentration throughout the trial. The increase in ergot alkaloid consumption during period 1 resulted in greater differences in the phenotypic performance parameter, including average daily gain (ADG), body condition scores (BCS), body weight (BW), and rectal temperatures that were evaluated in this study. 

The difference in ADG and BCS in response to increased consumption of ergot alkaloids is similar to data reported Thompson et al. [18], which identified a direct negative correlation to an animal’s ADG when the pasture infection rate increased. Moreover, comparison of phenotypic performance parameters of animals exposed to ergot alkaloids (EI) to animals not exposed to ergot alkaloids (EN) demonstrated the severity of exposure throughout the trial. Additionally, sufficient body condition has been shown to be one of the most important traits to cattle reproductive efficiency in a cow–calf herd, and decreases as a result of endophyte-infected tall fescue consumption [19]. 

Aside from animal growth performance, issues with thermotolerance have also frequently been seen in animals suffering from severe cases of fescue toxicosis [20,21]. Many of the symptoms of fescue toxicosis (ergot alkaloid exposure) are amplified during a period of heat stress, and the greatest loss in animal performance and consequently increased production losses occur during the summer months when grazing endophyte infected tall fescue [22]. It has been speculated that heat tolerant (i.e., Bos indicus) breeds of cattle have improved resistance to fescue toxicosis [10,23]. Several studies, including Poole et al. [8], have demonstrated that cattle with adaptions to manage heat stress perform better when exposed to ergot alkaloids and increased environmental temperatures. However, there is conflicting data that report no difference in growth rate [24], hormone concentration [23], or milk production [11] between Bos indicus and Bos taurus cattle grazing endophyte-infected tall fescue. Therefore, it remains unknown if the traits for heat tolerance and tolerance to ergot alkaloids are synonymous. 

Correlations have been previously made in which cattle that shed their winter hair coats earlier in the fescue season suffer less from the negative effects of fescue toxicosis [13]. In this study, weekly hair shedding scores (HSS) and hair coat scores (HCS) were taken to evaluate differences based on tolerance designation week to week, as well as initial rate of shedding. Both measures were significantly different between tolerant and susceptible animals grazing the endophyte-infected tall fescue. Both HCS and HSS were similar at the start of the study, and deviation between EI-TOL and EI-SUS animal occurred at week three of the study and continued through week seven (P-1). Interestingly, HSS and HCS re-converged to similar numeric values when ergot alkaloid concentrations decreased, yet ambient temperatures and humidity remained elevated consequentially when prolactin concentrations increased. Decreases in serum prolactin have been associated with fescue toxicity, and are often used as an indicator of ergot alkaloid exposure in cattle [25]. However, increased prolactin concentrations are associated with increasing day lengths and ambient temperatures, and initiate hair shedding [26,27]. Prolactin secretion has been linked with changes in environmental temperature, such that prolactin concentrations are elevated during warmer verses cooler months [28,29], and has been shown to regulate hair shedding [30,31]. It has been speculated that fescue toxicosis-induced hypoprolactinemia prevents shedding of the winter hair coat, therefore, cattle have an elevated body temperature and increased vulnerability to heat stress [32]. In the current study, EI-TOL animals had a tendency for lower serum prolactin concentrations when compared to EI-SUS animals as determined by the FTSM, thus, it can be assumed that those animals deemed tolerant had sufficient concentrations of serum prolactin to initiate hair shedding. Based on the other physiological measurements, the cattle in this study displayed many other symptoms of fescue toxicosis, and the lack of hypoprolactinemia was unexpected. The interaction of increased THI and exposure to ergot alkaloids impacts the severity of fescue toxicosis (reviewed by one), and the relatively low inclusion rate of the ergot alkaloids in the infected pastures may have altered serum prolactin concentrations. With EI-TOL animals having lower average serum prolactin concentrations than EI-SUS animals, it is hypothesized that these cattle were genetically predisposed to eliciting a shedding response at lower thresholds of prolactin. Using the same animals from this study, Koester et al. [33] showed that EI-TOL and EI-SUS animals have distinct fecal bacterial and fungal communities, further supporting that the proposed FTSM also results in differences in the microbiome of animals under FT stress. This classification using FTSM is further supported by the differences in T-Snip genotypes between EI-TOL and EI-SUS presented in the current study. These hypotheses build from the speculation of Aiken et al. [32] that genetic predispositions exist for necessary prolactin levels to initiate shedding.

More recent studies in various livestock species have reported that immune parameters have moderate to high heritability, as well as high genetic correlation with reproductive performance (lowly heritable traits), indicating that immune parameters can be used as a genetic trait for selecting animals that are resistant to specific diseases [34]. Using the protein array approach, numerous growth factors, chemokines, cytokine receptor antagonists, and anti-inflammatory and pro-inflammatory cytokines were examined during ergot alkaloid exposure. One of the major outcomes of this analysis is that many of the factors examined were significantly different or showed a statistical tendency to be higher at UPRS compared to BBCFL (Table 5 and Table 6). While cytokine differences between locations was not the primary focus of this study, these data demonstrate the immune system’s responsiveness to varying ergot alkaloid exposure, and warrants further investigation with known concentrations of ergovaline to better understand this interaction. Based on these data, it is hypothesized that cattle at the UPRS location experienced a greater immune response in response to higher ergot alkaloid exposure. A similar report described a hyperactive innate immune response, which may lead to an immuno-compromised animal in stocker steers when chronically exposed to ergovaline [35]. 

In the current study, vascular endothelial growth factor A (VEGFA) and G Protein-Coupled Receptor Associated Sorting Protein 1 (GPRASP-1) concentrations were greater in EI-TOL animals compared to EI-SUS animals. G Protein-Coupled Receptor Associated Sorting Protein 1 has been associated with downregulation of a variety of G Protein-Coupled receptors, including the D2-dopamine receptor, through lysosomal degradation. The D2-dopamine receptor has been shown to play a direct effect in cattle prolactin secretion, and is also involved in ergot alkaloids exposure’s decrease in prolactin secretion [36,37]. Prolactin is decreased when dopamine binds to the D2-dopamine receptor, and the cyclic ring structure of ergovaline closely mimics the ring structure of dopamine, allowing ergovaline to bind the D2-dopamine receptor and thus inhibit prolactin secretion [38,39]. In the current study, EI-TOL had greater GPRASP-1 concentrations, potentially causing the D2-dopamine receptor to be downregulated at a greater rate. This decrease in the prevalence of the D2-dopamine receptor decreased the opportunity for ergovaline to bind and decrease prolactin secretion, giving EI-TOL animals an advantage when grazing endophyte-infected tall fescue. Furthermore, this could contribute to EI-TOL animals having greater sensitivity to prolactin through downregulation of the entire prolactin secretion pathway, resulting in lower biological thresholds needed to initiate the biological roles of the hormone, which was previously discussed. 

Vascular endothelial growth factor A is a known vasodilator, and has been shown to increase micro-vascular permeability. In addition, it is known to play a particularly important role in vascular endothelial cells, where higher concentrations increase vascular permeability [40] and could provide protective effects against ergot alkaloid exposure. Vasoconstriction caused by exposure to ergot alkaloids further compounds the issues associated with fescue toxicosis, such as its ability to decrease the animal’s capacity for evaporative cooling [41]. Increased VEGFA concentrations and lower rectal temperature observed in the EI-TOL animals provide evidence that the EI-TOL animals selected using the FTSM have a greater ability to avoid heat stress and the vasoconstrictions effects of fescue toxicosis compared to EI-SUS animals. Several studies have examined changes in VEGFA to heat stress, and have focused on tissue and/or cellular responsiveness as opposed to whole animal changes. Vascular endothelial growth factor concentrations in the blood of heat tolerant (Bos Indicus) cattle compared to cattle that are more sensitive to heat stress (Bos Taurus) has not been directly investigated; however, Jyotiranjan et al. [42] and Iqbal et al. [43] reported increases in VEGF gene expression in response to thermal stress in goats and Bos indicus cattle breeds, respectively. Interestingly, of the eight Bos indicus breeds examined, only two breeds (Bhagnari and Lohani) displayed increased VEGF expression, but the authors indicate that this increase in VEGF is associated with adaptation to high altitude as opposed to heat tolerances [43]. Additionally, Jones et al. [44] and Aiken et al. [45] have speculated that decreases in serum progesterone concentrations could be caused by vasoconstriction of blood flow to the ovary in cattle consuming ergot alkaloids. Poole and colleagues [7] confirmed that ergovaline exposure reduced the diameter of the ovarian artery, leading to the functional corpus luteum, thus decreasing circulating progesterone concentrations. In the current study, EI-TOL animals tended to have higher progesterone concentrations compared to EI-SUS animals, which may be linked to greater ovarian artery diameter due to greater concentrations of circulating VEGFA in these animals. Thus, the EI-TOL animal’s ability to synthesize higher concentrations of VEGFA may provide the capacity to increase vessel diameter, which would result in substantial mitigation of the vasoconstriction seen with fescue toxicosis. Ultimately, further investigation is needed to evaluate these cytokines and their role in fescue toxicosis, as well as the tolerant animal’s ability to mount a stronger immune response to mitigate some of the negative effects of fescue toxicosis. 

Taken together, the selection strategy used in this study (i.e., FTSM) was showed to be effective in identifying groups of individuals expressing contrasting responses to ergot alkaloid exposure, i.e., fescue toxicosis [33]. Nonetheless, three items must be further discussed: first, the selection of these animals was based solely on their (adjusted) phenotypic performance. Although the T-Snip data presented in this study support that TOL animals have greater genetic tolerance to ergot alkaloid exposure than SUS animals, the accuracy of identifying individual differences from ergot alkaloid exposure using this test was not 100% [5]. Hence, there is still genetic variation in the animal’s response to ergot alkaloid exposure that could be explored. One way of getting ahold of individual genetic variation is through using, for example, the expected progeny differences (EPDs) for the growth rate of these animals as part of the selection criteria. However, there are some limitations on using the current EPDs for this purpose. These EPDs are based on a nationwide genetic evaluation (American Angus Association; http://www.angus.org/), which does not take into consideration the presence of GxE, which seems to be the case for ergot alkaloid exposure and for other stress-related traits [46]. Thus, the use of EPDs for this purpose may bias the selection of animals with greater tolerance to ergot alkaloid exposure. Although no source of genetic information was used in our selection process, it was expected that our approach captured part of the genetic potential of these animals. Assuming a) unbiased estimates for the fixed effects and b) independence between fixed and random effects included in the statistical model used for selection of TOL and SUS animals (see details in Koester at al. [33]), the estimated residuals that were used for classification of TOL and SUS animals should include both genotypic effects (additive and non-additive) and the true residual effects. Thus, assuming at least a moderate narrow-sense heritability for growth rate under ergot alkaloid exposure, it is expected that the estimated residuals include a substantial contribution of additive genetic values. Hence, although the selection process used in this study did not include prior genetic information from these animals, the data presented in this study demonstrate that TOL and SUS animals differed, at some level, in their genetic potential when exposed to ergot alkaloids. 

Additionally, another consideration is the potential confounding between the growth rate in the presence or absence of ergot alkaloids. In this study, it was assumed that animals expressed their growth as a function of response to ergot alkaloid exposure. In fact, the difference in results obtained between the two locations with endophyte-infected (toxic) fescue suggests that some minimum concentrations of ergot alkaloids are needed for animals to express a tolerant-related phenotype. In order to obtain accurate information on the relationship between the growth rate in the presence or absence of ergot alkaloids at the genetic level, a much larger sample size, with animals from a substantial number of sires represented across locations with varying concentrations of ergot alkaloids, would be needed to estimate the genetic correlation between the growth rate in the presence or absence of ergot alkaloids. Albeit needed, such a scenario would be difficult to achieve in a timely manner. At the phenotypic level, the use of biomarkers for ergot alkaloid exposure, such as prolactin [25] or those identified in our study (e.g., VEGFA), could provide additional information on the level of ergot alkaloid exposure on animals. This information could be used to evaluate whether a significant physiological response to ergot alkaloid exposure was observed in each animal, potentially allowing for better separation between animals showing a growth rate in the presence or absence of ergot alkaloids.

Lastly, our method was unidimensional, using only growth rate data for the selection of TOL and SUS animals. Hence, the evaluation of a fescue toxicosis selection index using multiple parameters and including other sources of information could be beneficial for more accurate selection of animals for response to ergot alkaloid exposure. Additional sources of information include the use of T-Snip, the biomarkers identified in this population (this study and Koester et al. [33]), genotypes at the dopamine receptor D2 gene locus [14], and more. Opportunities exist for the identification of additional biomarkers based on response-related traits other than growth rate, as used in this study. There is also a need for large-scale genomic studies for the identification of SNPs associated with variation in ergot alkaloid exposure related traits, which could be used to better identify animals with different genetic potential for fescue toxicosis. 

## 4. Conclusions

Taken together, these data provide support to validate the fescue toxicosis selection method (FTSM) proposed in this study through accurate collection of phenotypic performance parameters in a population of cattle chronically exposed to ergot alkaloids, and provide evidence to warrant additional analysis on the impact of ergot alkaloids on immune responsiveness in cattle that experience fescue toxicosis. Given the increased performance and hair shedding ability, greater hormone regulation and efficiency, and stronger cytokine responses, EI-TOL animals have clear mechanisms available to provide these advantages that can and should be selected for in animals raised in a fescue-dominant environment. 

## 5. Materials and Methods 

### 5.1. Animal Usage

This study was conducted at two locations in the piedmont of North Carolina, which were the Butner Beef Cattle Field Laboratory (BBCFL) in Bahama, NC and the Upper Piedmont Research Station (UPRS) in Reidsville, NC. All animal procedures were approved by the North Carolina State University Institutional Animal Care and Use Committee (NCSU IACUC #13-093-A; #17-043-A). Approval for animal use from the University Institutional Animal Care and Use Committee was on 19 August 2013 (#13-093-A) and was renewed and approved on 23 March 2017 (#17-043-A).

#### Animal Management

**Figure 3:**
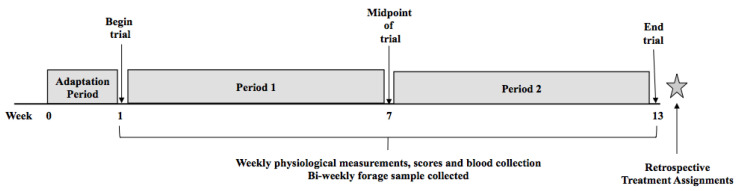
Experimental timeline used during cow performance phase of data collection from late-April to late-July 2016.

Cow performance and forage data were collected from late April to late July 2016 (Figure 3) when ergot alkaloid concentrations are greatest. Purebred Angus cows (*n* = 148) that ranged from two to four years of age all grazed endophyte-infected tall fescue during the entire experimental period. Cattle selected for this study were confirmed pregnant at 30 days post artificial insemination via ultrasonography, and were approximately 85 days post-conception at the start of the study. Additionally, cows selected for this study were weaned two weeks prior to the start of the study to remove the effect of lactation on animal performance. Cattle were offered ad libitum water and free choice minerals throughout the duration of the study, in addition to natural shade structures within the pastures. Additionally, a subset of cows (*n* = 27) were maintained under the same conditions on novel endophyte fescue pastures (non-toxic, EN) at the BBCFL to serve as a representative control group. Body weight (BW), body condition scores (BCS, as adapted from Richards et al., [47]), hair shedding score (HSS, as adapted from [13]), hair coat score (HCS, adapted from [48]), rectal temperatures, and jugular blood samples were collected weekly to evaluate the animal’s physiological response to ergot alkaloid exposure. Objective scores of BCS, HSS, and HCS were all collected by two trained evaluators and composited for an average score for each animal. Blood samples were collected via jugular venipuncture using 20-gauge needles and sterile 10.0 mL vacutainer tubes that contained no additive (Becton Dickerson, Franklin Lakes, NJ, USA). All blood samples were immediately placed on ice and then transported to the laboratory for processing. Whole blood samples were centrifuged the afternoon following collection each week for 25 min at 1500× *g* at 4 °C, then serum was drawn from vacutainer tubes and aliquoted into a glass dram vial and a plastic micro-centrifuge tube and stored at −80 °C until progesterone, prolactin, and cytokine analyses were conducted. 

### 5.2. Fescue Toxicosis Selection Method (FTSM)

**Figure 4:**
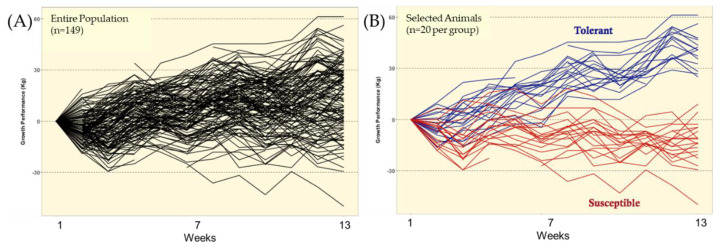
Graphical representation of the Fescue Toxicosis Selection Method (FTSM) to select the top 20 tolerant (EI-TOL) and 20 susceptible (EI-SUS) animals for further investigation within the entire population. (**A**) Displays the ranking of growth performance for the entire population (*n* = 149) observed over the 13-week period. (**B**) Displays the deviation in growth performance between the tolerant (*n* = 20) and susceptible (*n* = 20) animals over the 13-week period.

The classification of animals into tolerance (TOL) and susceptible (SUS) using the proposed Fescue Toxicosis Selection Method (FTSM) is fully described in Koester et al. [33]. In summary, growth data on each animal (Figure 3) was used to estimate the slope of the regression analysis of BW on weeks (average weekly gain; AWG, Figure 4A). This was performed based on three window periods: weeks 1 through 13 (ES), weeks 1 through 7 (P-1), and weeks 7 through 13 (P-2) to assess the effect of the increase in temperature from April to July, availability of forage (see Supplementary Table S1), and exposure of infected tall fescue (Table 1). The residuals from the analysis of AWG in a model including the fixed effects of location, parity, and initial body weight (covariate) were used to identify the window period in which the variation on the data was the largest, indicating the impact of the syndrome [46]. Results from this analysis indicated that data from P-1 resulted in the greatest residual variance [33]. Thus, the forty selected animals, those with the 10 most positive and 10 most negative residuals at each location, representing the TOL and SUS groups, respectively, from P-1 were used to evaluate performance and resistance to fescue toxicosis (Figure 4B).

### 5.3. T-Snip Genotyping

A detailed description of the genotyping of animals in this study for T-Snip (AgBotanica, LLC, Columbia, MO, USA) can be found in Galliou et al. [5]. In summary, blood cards were collected on each animal and shipped to GeneSeek (Neogen Genomics, Lincoln, NE, USA) for T-Snip genotyping. There were two, one, six, and one animals from EI-TOL at BBCFL for genotypes 1 to 4, respectively. The distribution of these T-Snip genotypes was one, five, three, and one, respectively, for EI-SUS at BBCFL; one, one, six, and two, respectively, for EI-TOL UPRS; and four, two, three, and zero, respectively, for EI-SUS UPRS cows.

### 5.4. Forage Management

Cattle at both locations grazed endophyte-infected tall fescue (toxic) pastures throughout the 13-week study. Cattle were rotationally grazed every two weeks at each location to ensure sufficient forage utilization and availability. Composite forage samples were taken from each pasture every two weeks to evaluate the nutrient quality and percentage of forage species available. Forage samples were clipped from approximately 20 locations in each pasture and composited. Fescue tiller samples were collected in November of 2016 to evaluate the pasture endophyte infection rate of the fescue. 

Nutrient quality samples were submitted within 24 h of collection for nutrient content, and then the results were averaged by experimental period (North Carolina Department of Agriculture Forage Laboratory, Raleigh, NC; USA, see Supplementary Table S1). From the same composite samples, forage was hand-separated in house by trained technicians to determine the percentage of fescue in relation to other various forage species (Table 3) making up forage dry matter. Collected fescue tiller samples were collected, rinsed, and shipped on ice to determine the pasture infection rate, and the average infection rate is reported by experimental period (Agrinostics Ltd. Co., Watkinsville, GA; USA, Table 3). Subsequent HPLC analysis of the forage samples for ergot alkaloid concentrations (MU Veterinary Medical Diagnostic Lab; [9]) demonstrated the change in ergovaline concentrations between locations and over the course of the grazing period (Table 3). 

### 5.5. Serum Assays and Analysis

Serum progesterone concentrations (P4) were analyzed on the FTSM, EI-TOL (*n* = 20), and EI-SUS (*n* = 20) animals for both locations at weeks 1, 3, 5, 7, 9, 11 and 13, which represent a bi-weekly measure during the entirety of the collection period. Concentrations were determined by a commercially available radioimmunoassay, the Immuchem Coated Tube Progesterone I125 RIA assay (ICN Pharmaceuticals, Inc., Costa Mesa, CA), as previously described by Lyons et al. [49]. Concentrations are reported in ng per mL, and the interassay and intraassay variation was 7.4% and 4.3%, respectively.

Serum prolactin concentrations (PRLs) were analyzed on the FTSM, EI-TOL (*n* = 20), and EI-SUS (*n* = 20) animals for both locations at weeks 1, 7, and 13, which represent the beginning, midpoint, and end of the collection period. Concentrations were determined by a commercially available Bovine Prolactin ELISA assay (MyBioSource, San Diego, CA, USA), as previously described by Poole et al. [8]. Concentrations were reported in ng per mL, and the interassay and intraassay variation was 11.7% and 4.2%, respectively.

Serum cytokine concentrations were analyzed on the FTSM, EI-TOL (*n* = 20), and EI-SUS (*n* = 20) animals for both locations at weeks 1, 7, and 13, which represent the beginning, midpoint, and end of the collection period. Cytokine concentrations were determined using a commercially available Bovine Cytokine Array (Quantibody^®^ Cytokine Arrays; RayBiotech, Inc., Peachtree Corners, GA, USA). The Q1 array permitted detection of interferon alpha (IFNAR1), interferon gamma (IFNG), interleukin (IL)-13, IL-1A, IL-36Ra, IL-21, chemokine CXCL10, CXCL9, CCL4, and tumor necrosis factor alpha (TNF), whereas the Q2 permitted detection of fibroblast growth factor (FGF)1, FGF1, G Protein-Coupled Receptor Associated Sorting Protein (GPRASP)-1, insulin like growth factor (IGF)-1, IL-2, IL-4, IL-15, CCL2, neural cell adhesion molecule 1 (NCAM1), and vascular endothelial growth factor (VEGFA). All bovine cytokine arrays were conducted according to the manufacturer’s recommendations. Following processing, all slides were returned to the manufacturer for laser scanning and data extraction. From the absorbance values provided by the manufacturer, serum concentrations were determined using a software (Q-Analyzer Software for QAB-CYT; RayBiotech, Inc., Peachtree Corners, GA, USA), and concentrations are reported in ng per mL for each individual cytokine. 

### 5.6. Statistical Analysis 

Performance and cytokine data were analyzed using the MIXED procedure of SAS 9.3 [50] with repeated measures. The individual animal was utilized as the experimental unit and the model for body condition scores, positive change in body condition scores, body weight, average daily gain, rectal temperature, hair coat score, hair shedding score, and hormone concentrations, and included Fescue Toxicosis Selection Method outcome (EI-TOL vs. EI- SUS), location (BBCFL vs. UPRS), trial period (P-1, P-2, and ES), and all respective interactions. For cytokine response data analysis, the individual animal was utilized as the experimental unit and the model for each cytokine response included treatment (EI-TOL vs. EI- SUS), location (BBCFL vs. UPRS), and time (Weeks 1, 7, and 13). The distribution of T-Snip genotypes between EI-TOL and EI-SUS was analyzed using a logistic multinomial model using the T-Snip genotype of animals as the response variable and the FTSM group (EI-TOL and EI-SUS) as a fixed effect in the model. This analysis was carried out in R [51]. Results were recorded as least square means ± SEM, where statistical significance was reported at *p* ≤ 0.05 and a statistical tendency at 0.05 ≤ *p* ≤ 0.10. 

## Figures and Tables

**Table 1:** Endophyte-infected tall fescue characteristics.

Forage Percentage	BBCFL ^1^			UPRS ^2^				Combined Locations			
	P-1 ^3^	P-2 ^4^	ES ^5^	P-1 ^3^	P-2 ^4^	ES ^5^	P-1 ^3^		P-2 ^4^	ES ^5^	
Percent fescue (%)	72.5	56.0	64.3	72.9	63.3	68.1	72.7		59.7	66.2	
Tiller infection ^6^ (%)	84.2	77.5	80.8	82.5	90.0	86.3	83.3		83.8	83.5	
Ergot alkaloid analysis ^7^											
Ergosine (μg/kg)	– ^8^	–	–	–	333.3 ± 322	166.7 ± 230	–		166.7 ± 230	83.3 ± 163	
Ergotamine (μg/kg)	–	–	–	–	175.0 ± 202	87.5 ± 44	–		87.5 ± 142	43.8 ± 101	
Ergocornine (μg/kg)	–	–	–	–	53.3 ± 61	26.7 ± 43	–		26.7 ± 43	13.3 ± 30	
Ergocryptine (μg/kg)	–	–	–	–	150.0 ± 93	75.0 ± 66	–		75.0 ± 66	37.5 ± 47	
Ergocristine (μg/kg)	–	–	–	–	48.3 ± 51	24.2 ± 36	–		24.2 ± 36	12.1 ± 25	
Ergovaline (μg/kg)	263.3 ± 52	106.7 ± 41	185.0 ± 38	436.7 ± 83	196.7 ± 102	316.7 ± 64	350 ± 58		151.7 ± 55	250.8 ± 42	
Total (μg/kg)	263.3 ± 52	106.7 ± 41	185.0 ± 38	436.7 ± 83	956.7 ± 43	696.7 ± 45	350.0 ± 58		531.7 ± 25	440.8 ± 37	

^1^ BBCFL: Butner Beef Cattle Field Laboratory location, Bahama, NC; ^2^ UPRS: Upper Piedmont Research Station location, Reidsville, NC; ^3^ P-1: Period 1, using values from weeks one, three, and five; ^4^ P-2: Period 2, using values from weeks 7, 9 and 11; ^5^ ES: Entire study, using values from weeks 1, 3, 5, 7, 9, and 11; ^6^ Phytoscreen^©^ immunochemical endophyte test, Agrinostics Ltd. Co., Watkinsville, GA, USA; ^7^ HPLC analysis at the University of Missouri Veterinary Medical Diagnostic Laboratory (Columbia, MO;USA) [9]; ^8^ Denotes not detected via HPLC.

**Table 2:** Expected proportion of T-Snip genotypes ^2^ between tolerant (TOL) and susceptible (SUS) cows in the endophyte-infected (EI) fescue locations.

FTSM ^1^	T-Snip Genotypes			
	1	2	3	4
TOL	0.150 ^a^ (0.08)	0.100 ^a^ (0.07)	0.600 ^a^ (0.11)	0.15 ^a^ (0.08)
SUS	0.263 ^a^ (0.10)	0.368 ^b^ (0.11)	0.316 ^b^ (0.11)	0.05 ^a^ (0.05)

^1^ FTSM: Fescue toxicosis selection method group. There was an effect (*p* = 0.089) of FTSM on the distribution of T-Snip genotypes; ^2^ Values of T-Snip (AgBotanica, LCC, Columbia, MO, USA) genotypes range from 1 to 5, with expected greater tolerance to fescue toxicosis in animals with greater values; ^a,b^ Expected proportions within a T-Snip lacking the same superscript are statistically different from each other (*p* < 0.05).

**Table 3:** Performance parameters for Angus cows consuming either novel endophyte (EN) or endophyte-infected (EI) fescue from late April to late July 2016.

Item	Treatment ^1,2^			SEM	*p* Value
	EN	EI-TOL	EI-SUS		
Initial BW, kg	512.1	501.4	518.6	10.6	0.5082
Final BW, kg	576.0 ^a,^*	546.6 ^a,b,^*	518.3 ^b^	12.3	0.0004
ADG, kg/d	0.703 ^a^	0.497 ^b^	−0.003 ^c^	0.05	<0.0001
BCS ^3^	6.09 ^a^	5.50 ^b^	5.59 ^b^	0.06	<0.0001
HCS ^4^	1.89 ^a,^*	1.98 ^a,^*	2.34 ^b^	0.04	<0.0001
HSS ^5^	1.99 ^a^	2.36 ^b^	2.64 ^c^	0.04	<0.0001
Rectal temp., °C	38.6 ^a^	38.7 ^b^	38.9 ^c^	0.02	<0.0001

^a–c^ Within the row, means without a common superscript significantly differ (*p* ≤ 0.05); * 0.05 < *p* ≤ 0.10 determined a statistical tendency; ^1^ Values are reported as least square means for the experiment; ^2^ EN: cattle grazing novel endophyte fescue (nontoxic, *n* = 27); EI-TOL: cattle grazing endophyte-infected fescue deemed tolerant through FTSM (*n* = 20); EI-SUS cattle grazing endophyte-infected fescue deemed susceptible through FTSM (*n* = 20); ^3^ BCS = body condition score (1–9 scale); ^4^ HCS = hair coat score (1–5 scale); ^5^ HSS = hair shedding score (1–5 scale).

**Table 4:** Performance parameters for Angus cattle grazing endophyte-infected fescue deemed either tolerant (EI-TOL) or susceptible (EI-SUS) through FTSM from late April to late July 2016.

Time	P-1 ^1^				P-2 ^2^				ES ^3^			
Treatment ^4,5^	EI-TOL	EI-SUS	SEM	*p*-Value	EI-TOL	EI-SUS	SEM	*p* Value	EI-TOL	EI-SUS	SEM	*p*-Value
ADG, kg/d	1.8 ^a^	−0.55 ^b^	0.13	<0.0001	0.5	0.5	0.2	0.8726	1.2 ^a^	−0.025 ^b^	0.1	<0.0001
∆BCS ^6^	0.488 ^a^	0.013 ^b^	0.09	0.0008	0.613 *	0.363 *	0.09	0.0625	1.05 ^a^	0.375 ^b^	0.10	<0.0001
∆HCS ^7^	−2.013 ^a^	−0.913 ^b^	0.174	<0.0001	−0.300 ^a^	−1.150 ^b^	0.207	0.0062	−2.313	−2.063	0.226	0.4398
∆HSS ^8^	−1.925 ^a^	−0.613 ^b^	0.198	<0.0001	−0.763 ^a^	−1.838 ^b^	0.202	0.0006	−2.688	−2.450	0.248	0.5027
∆RT ^9^, °C	−0.250	0.105	0.176	0.1630	0.505	0.230	0.126	0.1299	0.250	0.325	0.179	0.7680

^a,b^ Within the row, means without a common superscript significantly differ (*p* ≤ 0.05); * *p*-values 0.05 < *p* ≤ 0.10 determined a statistical tendency; ^1^ P-1: Period 1, using data collected from weeks one, three, and five; ^2^ P-2: Period 2, using data collected from weeks 7, 9, and 11; ^3^ ES: Entire study, using data collected from weeks 1, 3, 5, 7, 9, and 11; ^4^ Values are reported as least square means for the experiment; ^5^ EI-TOL: cattle grazing endophyte-infected fescue deemed tolerant through FTSM (*n* = 20); EI-SUS cattle grazing endophyte-infected fescue deemed susceptible through FTSM (*n* = 20); ^6^ ∆BCS = change in body condition score (1–9 scale) over the given time frame; ^7^ ∆HCS = change in hair coat score (1–5 scale) over the given time frame; ^8^ ∆HSS = change in hair shedding score (1–5 scale) over the given time frame; ^9^ ∆RT = change in rectal temperature (°C) over the given time frame.

**Table 5:** Growth factors, chemokines, and cytokine receptor antagonists.

Treatment ^1,2^	EI-TOL						EI-SUS									
Location	BBCFL ^3^			UPRS ^4^			BBCFL ^3^			UPRS ^4^				*p*-Values		
Week	1	7	13	1	7	13	1	7	13	1	7	13	SEM	TRT	LOC	TIME
Growth factors ^5^																
FGF1	196.5 *	110.3 *	77.3 *	233.8 *	145.2 *	120.0 *	98.0 *	69.3 *	52.3 *	203.8 *	163.0 *	99.8 *	60.4	0.3381	0.0793	0.0716
FGF2	9.4	28.1	46.8	27.9	16.6	16.1	34.9	26.6	19.9	25.0	1937	7.3	14.8	0.8324	0.3327	0.9849
IGF1	6.8	21.3	10.4	21.2	4.3	6.5	18.7	14.1	3.9	10.2	8.8	9.3	4.2	0.7330	0.3602	0.1441
VEGFA	572.64	429.9	441.0	1117.2	512.4	711.2	208.7	152.9	220.1	253.64	769.8	297.2	114.2	0.0444	0.1070	0.8319
Chemokines ^5^																
CCL2	856.4	2817.6	269.7	2012.2	1946.2	1979.8	269.2	2124.8	277.7	1022.8	1622.4	1374.8	898.7	0.3210	0.2984	0.1452
CCL4	8.7	11.9	141.3	16.8 ^a^	616.7 ^b^	754.9 ^b^	9.8	10.8	191.8	7.6 ^a^	171.1 ^a,b^	404.4 ^b^	192.7	0.2831	0.0245	0.0428
CXCL9	35.7	41.6	73.2	41.8	250.9	220.1	98.2	42.2	62.8	75.9	56.3	150.1	70.2	0.4644	0.0708	0.4237
CXCL10	34.7	44.3	68.1	45.1	91.0	104.6	42.2	34.9	57.5	80.8	54.2	67.1	27.7	0.5909	0.0929	0.4521
Cytokine receptors antagonists ^5^																
IL36RN	69.2	94.2	167.4	109.1	562.9	512.9	67.8	73.8	110.0	205.8	252.2	357.6	136.7	0.3590	0.0044	0.1922
GPRASP-1	4.1 ^a^	6.1 ^b^	4.2 ^a^	4.2	4.7	4.7	3.8 ^a^	5.2 ^b^	3.8 ^a^	4.2	4.0	3.8	0.4	0.0343	0.3268	0.0033
NCAM1	33.8	33.7	30.7	33.1	32.3	42.4	29.9	31.9	26.9	30.6	35.0	39.4	3.8	0.3428	0.0496	0.5422

^a,b^ Within the row, means without a common superscript significantly differ (*p* ≤ 0.05); ^*^ *p*-values 0.05 < *p* ≤ 0.10 determined a statistical tendency; ^1^ Values are reported as least square means for the experiment; ^2^ EI-TOL: cattle grazing endophyte-infected fescue deemed tolerant through FTSM (*n* = 20); EI-SUS cattle grazing endophyte-infected fescue deemed susceptible through FTSM (*n* = 20); ^3^ BBCFL: Butner Beef Cattle Field Laboratory location, Bahama, NC; ^4^ UPRS: Upper Piedmont Research Station location, Reidsville, NC; ^5^ Growth factors, chemokines, and cytokine receptors antagonist concentrations reported as ng/mL.

**Table 6:** Anti-inflammatory and pro-inflammatory cytokines and reproductive hormones.

Treatment ^1,2^	EI-TOL							EI-SUS									
Location	BBCFL ^3^			UPRS ^4^			BBCFL ^3^			UPRS ^4^				*p*-Values		
Week	1	7	13	1	7	13	1	7	13	1	7	13	SEM	TRT	LOC	TIME
Anti-inflammatory ^5^																
IL4	89.7	256.8	30.1	286.1	215.8	407.7	48.9	47.8	26.4	198.2	110.1	95.2	133.9	0.1141	0.0909	0.9801
IL13	6.7	7.4	5.0	13.1	9.5	10.1	9.5	10.7	2.1	26.1	10.4	6.0	5.1	0.4710	0.0672	0.1021
Pro-inflammatory ^5^																
IFNAR1	20.0 ^a^	44.8 ^a^	460.2 ^b^	71.5 ^a^	304.5 ^b^	518.2 ^b^	44.4^a^	52.4^a^	362.7 ^b^	29.2 ^a^	259.9 ^b^	306.5 ^b^	49.5	0.3921	0.2361	0.0001
IFNG	1.7	1.8	1.8	2.5	3.2	4.5	3.9	2.0	1.2	4.3	3.5	2.9	1.5	0.6638	0.1083	0.8552
IL1A	72.0	53.3	209.0	99.2	273.7	380.6	146.0	65.6	116.6	162.4	159.3	266.7	85.1	0.5768	0.0287	0.1060
IL15	13.1	41.5	4.9	25.8	22.3	32.6	9.7	14.7	6.1	12.6	30.1	11.5	12.7	0.1847	0.2822	0.2296
IL2	107.6	218.8	255.0	100.4	191.0	87.8	216.0	110.1	143.8	30.6	43.1	60.0	93.8	0.2878	0.1113	0.9128
IL21	60.9	54.0	92.4	89.2	177.5	178.2	118.1	68.3	48.7	167.7	66.4	132.6	58.6	0.8095	0.0795	0.8667
TNF	43.0	52.0	77.3	86.3	175.5	263.5	113.9	64.2	53.1	176.4	64.2	130.4	70.5	0.7046	0.0523	0.7093
Reproductive hormones ^5^																
Prolactin	55.0 ^a^	102.4 ^b^	74.5 ^a,b^	51.0 ^a^	166.0 ^b^	140.7 ^a,b^	89.5 ^a^	167.5 ^b^	105.7 ^a,b^	60.6 ^a^	217.6 ^b^	189.4 ^a,b^	38.8	0.0787	0.0918	0.0019
Progesterone	5.0^a^	2.3 ^b^	2.9 ^b^	2.9	1.8	3.0	3.8 ^a^	2.5 ^b^	3.1^a,b^	2.4	2.3	2.4	0.5	0.0895	0.0018	0.0003

^a,b^ Within the row, means without a common superscript significantly differ (*p* ≤ 0.05); * *p*-values 0.05 < *p* ≤ 0.10 determined a statistical tendency; ^1^ Values are reported as least square means for the experiment; ^2^ EI-TOL: cattle grazing endophyte-infected fescue deemed tolerant through FTSM (*n* = 20); EI-SUS cattle grazing endophyte-infected fescue deemed susceptible through FTSM (*n* = 20); ^3^ BBCFL: Butner Beef Cattle Field Laboratory location, Bahama, NC; ^4^ UPRS: Upper Piedmont Research Station location, Reidsville, NC; ^5^ Anti-inflammatory and pro-inflammatory cytokines and reproductive hormone concentrations reported as ng/mL.

## Supplementary Materials

The following are available online at www.mdpi.com/xxx/s1,Table S1. Nutritive Value (on a DM basis) of forage samples
