# Supplementary material for: Evaluation of Resistance to Fescue Toxicosis in Purebred Angus Cattle Utilizing Animal Performance and Cytokine Response
Source: Toxins (Basel). 2020 Dec 14;12(12):796. doi: 10.3390/toxins12120796 (PMC7764894; doi:10.3390/toxins12120796)
Supplement: Supplementary file 1 [file toxins-12-00796-s001.pdf]

Supplementary Materials: Evaluation of Resistance to Fescue Toxicosis in Purebred Angus Cattle Utilizing Animal Performance and Cytokine Response

Daniel H. Poole, Kyle J. Mayberry, McKayla Newsome, Rebecca K. Poole, Justine M Galliou, Piush Khanal, Matthew H. Poore and Nick V. L. Serão

Table S1. Nutritive Value (on a DM basis) of forage samples.

	BBCFL[1]			UPRS[2]			Combined Locations		
	**P-1[3]**	**P-2[4]**	**ES[5]**	**P-1[3]**	**P-2[4]**	**ES[5]**	**P-1[3]**	**P-2[4]**	**ES[5]**
DM (%)	24.6	35.6	30.1	29.7	29.7	29.7	27.2	32.7	29.9
CP (%DM)	9.98	9.45	9.72	10.3	10.5	10.4	10.2	10.0	10.1
NDF (%DM)	62.4	65.2	63.8	62.2	63.4	62.8	62.3	64.3	63.3
ADF (%DM)	36.0	39.8	37.9	37.6	38.7	38.2	36.8	39.3	38.0
TDN (%DM)	63.8	60.9	62.3	62.6	61.7	62.1	63.2	61.3	62.2
Ash (%DM)	6.04	6.06	6.05	5.79	6.13	5.96	5.91	7.00	6.01
Ca (%DM)	0.27	0.31	0.29	0.30	0.34	0.32	0.29	0.32	0.30
P (%DM)	0.20	0.18	0.19	0.22	0.24	0.23	0.21	0.21	0.21
Mg (%DM)	0.20	0.23	0.22	0.19	0.20	0.19	0.20	0.22	0.21
Na (%DM)	0.01	0.01	0.01	0.01	0.01	0.01	0.01	0.01	0.01
K (%DM)	2.08	1.65	1.87	2.09	2.06	2.08	2.09	1.85	1.97
Cu (ppm)	4.83	6.67	5.75	5.00	4.83	4.92	4.92	5.75	5.33
Fe (ppm)	78.00	140.2	109.1	64.33	97.33	80.83	71.17	118.8	94.96
Mn (ppm)	73.17	95.33	84.25	44.17	47.50	45.83	58.67	71.42	65.04
Zn (ppm)	23.33	27.83	25.58	18.50	17.50	18.00	20.92	22.67	21.79

[1] BBCFL: Butner Beef Cattle Field Laboratory location, Bahama, NC, [2] UPRS: Upper Piedmont Research Station location, Reidsville, NC, [3] P-1: Period 1, using average nutrient values from weeks 1, 3 and 5, [4] P-2: Period 2, using average nutrient values from weeks 7, 9 and 11, [5] ES: Entire Study, using average nutrient values from weeks 1, 3, 5, 7, 9, and 11.
